# Systematic review of the efficacy of yoga and mindfulness in the management of pediatric obesity

**DOI:** 10.1111/nyas.15245

**Published:** 2024-12-19

**Authors:** Mardia López‐Alarcón, Miguel A. Villasis‐Keever, José R. Fernández

**Affiliations:** ^1^ División de Investigación Clínica Instituto Mexicano del Seguro Social Mexico City Mexico; ^2^ Unidad de Investigación en Evaluación y Síntesis de la Evidencia, Coordinación de Investigación en Salud Instituto Mexicano del Seguro Social Mexico City Mexico; ^3^ Department of Nutrition Sciences and Department of Biostatistics University of Alabama at Birmingham Birmingham Alabama USA

**Keywords:** adolescents, efficacy, meditation, mindfulness, obesity, safety, yoga

## Abstract

The neuroplasticity of adolescents could make them responsive to interventions affecting brain maturation such as yoga and mindfulness. We aimed to determine their efficacy and safety for the management of children and adolescents with obesity. A systematic search using MEDLINE, EMBASE, and PsycInfo was performed up to March 2024. We considered randomized controlled trials (RCTs) using yoga or mindfulness alone (or combined with standard therapy) compared to placebo, nothing, or standard therapy for weight loss. Methodological quality of studies was assessed with the Risk of Bias 2 tool. The primary outcomes were changes in weight and adiposity (kg, body mass index [BMI], BMI *z*‐score, fat mass, waist circumference, waist‐to‐hip ratio). We assessed 4 yoga and 7 mindfulness RCTs, including 620 participants 8–19 years old. The number of participants varied per type of intervention (yoga, *n* = 10–63; mindfulness, *n* = 11–47). Comparators were no‐intervention or active controls. All yoga trials reported anthropometric improvements, but all trials combined yoga with extra physical activity. Five out of seven mindfulness trials reported anthropometric improvements. The methodological quality of the RCTs was low. No safety information was reported. The effect of yoga and mindfulness on psychological and metabolic variables was inconsistent. This evidence is insufficient to recommend yoga or mindfulness for the management of adolescents with obesity.

## Graphical abstract

The neuroplasticity of adolescents could make them responsive to interventions affecting brain maturation. Meditation‐based interventions like yoga and mindfulness appear to influence brain maturation processes. Yoga and mindfulness use meditation but have differences that might impact the weight of individuals with obesity through different mechanisms. Although yoga might have an impact via meditation and increased energy expenditure, mindfulness probably does so through meditation and modulating stress, anxiety, and appetite signals.

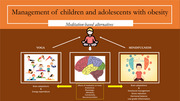

## INTRODUCTION

Obesity in pediatric populations has reached epidemic proportions worldwide. According to the World Health Organization, 158 million children and adolescents in the world presented with obesity in 2020, and it is predicted that this figure will increase to 253 million by 2030.^1^ Childhood obesity is associated with persistent obesity in adulthood and a high risk of chronic non‐communicable diseases, premature death, and disability later in life.^2^ In the short term, children and adolescents with obesity experience metabolic alterations, elevated markers of cardiovascular damage, joint injury, and different degrees of psychological and eating disorders that potentially interfere with their overall health^2^, ^3^ and obesity management.

Many of the psychological disorders observed in adolescents with obesity are likely associated with specific physiological maturation processes. In general, brain neurodevelopment continues during puberty, including the maturation of the dopaminergic pathways and the regulation of inhibitory and excitatory signaling, resulting in increased reward sensitivity and reduced behavioral control.^4^, ^5^, ^6^, ^7^ The last growth spurt that takes place during adolescence requires more energy to deposit new tissue, and therefore an increase in appetite is observed. Such increased appetite can result in hyperphagia.^7^, ^8^ Adolescence is also a stage of social maturation when children start feeling behavioral independence, including for food choices. These physiological features influence the probability for teenagers to select unhealthy, highly rewarding, palatable food to be consumed in excess, which may result in excessive weight gain and/or obesity.^6^, ^7^ In addition, children and adolescents with obesity frequently experience weight‐related bullying and teasing that lead to psychological manifestations including mood disorders, (e.g., weight stigma, poor self‐esteem, depression, anxiety, stress) and eating disorders, (e.g., binge eating disorder, night eating syndrome). These manifestations cause social isolation, rejection of treatment, and further weight gain.^7^, ^9^, ^10^, ^11^ Stress is particularly relevant because it has a bidirectional relationship with obesity; that is, although obesity promotes stress, it alters the hypothalamic–pituitary–adrenal axis, which consequently increases the susceptibility to gain weight.^12^ Thus, children and adolescents with obesity are more likely to engage in practices such as emotional eating and overeating and lower levels of physical activity, and they are also more likely to report higher rates of anxiety, depression, and sleep disturbances compared to their normal weight counterparts.^13^, ^14^, ^15^, ^16^

International organizations have recognized the need for behavioral interventions in addition to dietary and physical activity interventions to impact the emotional, psychological, and mental states of children and adolescents with obesity. As part of a multicomponent approach, the US Preventive Services Task Force recently recommended providing intensive behavioral interventions to children and adolescents with obesity for at least 26 contact hours for up to a year to promote improvements in weight status. This recommendation was founded on results of recent systematic reviews and meta‐analyses of randomized controlled trials (RCTs) of children and adolescents with obesity who received behavioral interventions, including motivational interventions, cognitive–behavioral interventions, health‐promoting education, interpersonal psychotherapy, coaching, and nutritional counseling and portion control.^17^, ^18^, ^19^

Neuroplasticity during childhood and adolescence may also offer an opportunity for improvement because children could be particularly responsive to interventions that impact the brain and provide the circumstances for strategies to manage stress and unhealthy habits.^20^ Meditation‐based interventions such as yoga and mindfulness appear to influence brain maturation processes, stress, and perhaps unhealthy behaviors. Therefore, yoga and mindfulness might be useful in managing obesity. Scientific evidence has demonstrated the positive effects of meditation on brain anatomy and function in both adults and adolescents,^21^, ^22^ but its effectiveness in obesity management has not been demonstrated. Although yoga and mindfulness use meditation, these techniques have important differences that might impact the body weight of individuals with obesity through different mechanisms. Although yoga might have an impact via meditation and increased energy expenditure,^23^, ^24^ mindfulness probably does so through meditation and by modulating stress, anxiety, appetite signals, and the inflammatory status that usually accompanies obesity.^25^, ^26^, ^27^, ^28^, ^29^

The practice of yoga in adults has been associated with mainly non‐serious musculoskeletal adverse events. Compared with other physical activity interventions, the risk of adverse events related to yoga was comparable; most were mild and transient.^30^ The more frequently reported adverse events associated with mindfulness in adults are anxiety, distress, and discomfort, but these unpleasant feelings are likely associated with the increased awareness of experiences, which is indeed the foundation of this technique.^31^ More severe adverse events, such as psychosis or mania, have been reported in participants with past mental health conditions and sleep deprivation.^32^ No studies conducted to assess yoga or mindfulness safety in children were identified.

In summary, the usefulness and safety of these meditation‐related interventions for the management of pediatric obesity need to be evaluated, particularly when considering the developmental characteristics of children and adolescents and outcomes of interest beyond anthropometric measurements.

### Objective

To determine the efficacy and safety of yoga and mindfulness for the management of children and adolescents with obesity.

## MATERIALS AND METHODS

This systematic review was performed following the Preferred Reporting Items for Systematic Reviews and Meta‐Analysis guidelines.^33^ It addresses the question of whether yoga and mindfulness are effective and safe for the management of obesity in children and adolescents.

### Search strategy

We searched for relevant RCTs in the following databases: MEDLINE (from inception to March 2024), EMBASE (from inception to March 2024), and PsycInfo (1980 to February 03, 2024) (see Supporting Information). We also searched previous systematic reviews/meta‐analyses and the bibliography of included articles.

We examined RCTs in which meditation, mindfulness, or yoga were assessed, either alone or in combination with standard therapy (e.g., low‐calorie diet, exercise, behavioral), compared with placebo, nothing, or standard therapy for weight loss. There was no restriction regarding length of intervention, follow‐up, or language of publications. RCTs including patients with eating disorders were excluded. In the case of RCTs with more than one publication, only the article containing the information on the primary outcomes was considered.

Included RCTs included children and/or adolescents with obesity. Participants were 19 years of age or younger and were of any sex or gender, national origin, or place of residence. There was no restriction on overweight/obesity definitions (as defined by study authors).

### Outcome measures

Changes in weight (kg, body mass index [BMI], BMI *z*‐score), adiposity (arm circumference, fat mass, body fat), and adiposity distribution (waist circumference, waist‐to‐hip ratio) after the intervention were considered primary outcomes.

Secondary outcomes included obesity‐associated comorbidities (other than non‐communicable diseases), hyperinsulinemia (as defined by authors), insulin resistance (as defined by authors), dyslipidemia (as defined by authors), and alterations in hunger or satiety (as defined by authors).

### Study selection and data extraction

Two of the authors (M.A.V.‐K. and M.L.‐A.) independently screened titles and abstracts and assessed full‐text articles for eligibility and extracted the relevant data from the included studies. Decisions were reached at each of these three phases when both evaluators agreed. Disagreements were solved by consensus.

### Risk of bias

The risk of bias of RCTs was assessed with the Risk of Bias 2 tool in accordance with Cochrane Handbook for Systematic Reviews of Interventions.^34^ Each quality component was classified as low, high, or some concerns of bias. We assessed randomization process, deviations from intended interventions, missing outcome data, measurement of the outcome, and selection of the reported results.

### Statistical analysis

For each included study, all relevant variables were collected and synthesized in evidence tables according to the intervention: yoga or mindfulness. Meta‐analysis was not performed due to discrepancies in the form and time of application of the interventions, populations studied (e.g., different ages, gender, types of yoga or mindfulness interventions), and outcome measures.

## RESULTS

**FIGURE 1:**
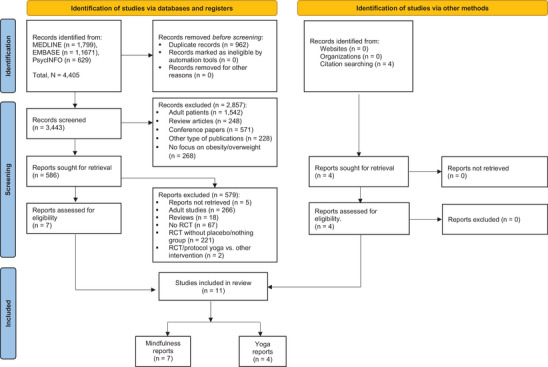
Flowchart for selecting randomized clinical trials of yoga, meditation, and mindfulness for managing pediatric obesity. RCT, randomized controlled trial.

Our search generated 3443 records after duplicates were removed. Eleven RCTs fulfilled selection criteria and were included in this review; four used yoga as an intervention and seven used mindfulness. No study claiming unspecified meditation as an intervention was identified. The study flow diagram is presented in Figure 1.

The 11 reviewed RCTs included 620 participants aged 8–19 years. The number of participants for each study varied per type of intervention (yoga 10–63, mindfulness 11–47). Variations in the length of follow‐up (yoga 8–18 weeks, mindfulness 6–24 weeks) and outcomes to evaluate effects in excess weight or adiposity (body weight, BMI, BMI *z*‐score, waist‐to‐hip ratio, fat mass, arm circumference) were also observed. Comparators were no‐intervention (waiting list or usual care) or active controls (comparable number of sessions). The influence of gender was analyzed in one study assessing yoga and one assessing mindfulness. None of the studies reported adverse events, but it is not clear whether they did not occur or were not assessed. Details about the quality of the reviewed trials are presented in Table 1. Most had problems in the randomization process, unblinded assessments, and attrition.

**TABLE 1:** Analysis of the included randomized control trials based on the risk of bias assessment.

Study	Randomization process	Deviations from intended interventions	Missing outcome data	Measurement of the outcome	Selection of the reported results	Overall
**Yoga**						
Sabet Sarvestani et al.^35^	Some concerns	Some concerns	Low risk	High risk	Low risk	Some concerns
Seo et al.^36^	High risk	High risk	Low risk	High risk	Low risk	High risk
Halperin et al.^37^	High risk	Some concerns	Low risk	High risk	Low risk	Some concerns
Jain et al.^38^	Low risk	High risk	High risk	High risk	Low risk	High risk
**Mindfulness**						
Barnes and Kristeller^43^	Some concerns	Some concerns	Some concerns	High risk	Low risk	Some concerns
Daly et al.^42^	Low risk	High risk	High risk	High risk	Low risk	High risk
Stavrou et al.^44^	Some concerns	Some concerns	Low risk	High risk	Low risk	Some concerns
Kumar et al.^41^	Low risk	Low risk	Low risk	Low risk	Low risk	Low risk
Emmanouil et al.^45^	High risk	Some concerns	Low risk	High risk	Low risk	Some concerns
Shomaker et al.^46^	Low risk	High risk	High risk	High risk	Low risk	High risk
López‐Alarcón et al.^47^	High risk	High risk	Some concerns	High risk	Low risk	High risk

### Yoga

Four RCTs assessing the effect of yoga in anthropometric measurements of children and adolescents with obesity were analyzed (Table 2).^35^, ^36^, ^37^, ^38^ All studies reported significant improvements in weight measurements in the groups exposed to yoga, but in all cases, yoga was combined with physical exercise other than yoga and another type of intervention, which were not offered to controls. A study that compared yoga combined with physical exercise at least three times per week and behavioral‐changing therapy against no‐intervention controls found decreases in BMI (−1.07 vs. 0.24 kg/m^2^, *p *< 0.001), body weight (−2.75 vs. 0.62 kg, *p *< 0.001), and arm circumference (−2.31 vs. 0.5 cm, *p* < 0.001) in the intervention. Increased anthropometric measurements were found in the control groups.^35^ A study that compared yoga plus physical exercise after classes and health education sessions—against no intervention—reported significant decreases in absolute and relative fat mass (−3.93 vs. −2.07 kg, *p* = 0.015; and −4.31% vs. −1.50%, *p* = 0.031), but not in BMI (−1.01 vs. 0.53 kg/m^2^, *p* = 0.17) in the intervention group compared to the control group.^36^

**TABLE 2:** Studies that analyzed the efficacy of yoga in the management of children and adolescents with obesity.

Author Country	Participants	Type of intervention (*n*)	Follow‐up	Anthropometric outcomes (delta)	Comments
Sabet Sarvestani et al.^35^ Iran	Girls 11–15 years BMI > 95th	Intervention: Yoga + behavioral therapy + exercise (*n* = 30) Control: Behavioral therapy (*n* = 30)	4‐h sessions 4 times per week, for 16 weeks, including 2 h of behavioral modification. Unclear if meditation was included in the intervention. Additional exercise at least three times a week Three 2‐h sessions for behavioral modification	Yoga vs. control Body weight, kg: −2.75 vs. 0.62, *p*<0.001 BMI, kg/m^2^: −1.07 vs. 0.24, *p*<0.001 Arm circumference, cm: −2.31 vs. 0.5, *p*<0.001	80% completed follow‐up
Seo et al.^36^ Korea	Boys 13–15 years BMI > 95th	Intervention: Yoga + health education + exercise (*n* = 10) Control: No intervention, indications for not exercising ≥30 min per week during study time (*n* = 10)	1‐h sessions of yoga asanas + 10 min relaxation 3 times per week, for 8 weeks. Physical exercise after classes Basal and final interviews. No after‐class sessions	Yoga vs. control BMI, kg/m^2^: −1.01 vs. 0.53, *p* = 0.17 Fat mass: −3.93 vs. −2.07 kg, *p* = 0.015 −4.31 vs. −1.50%, *p* = 0.031 Resting metabolic rate (kcal) increased in yoga group: 44.30 vs. 15.90, *p* = 0.195	All completed follow‐up
Halperin et al.^37^ Puerto Rico	Boys and girls 18–19 years BMI > 25	Intervention: Yoga + physical exercise (*n* = 19) Control: Standard care (*n* = 21)	75‐min sessions of Hatha yoga per week for 10 weeks + instructions for relaxation, meditation, and mindful eating. Follow‐up 6 months post‐intervention. No information	Yoga vs. control At week 10 BMI, kg/m^2^: −0.8 vs. 0.7, *p*<0.001 At 6 months: −1.2 vs. 0.8, *p*<0.001	All completed follow‐up
Jain et al.^38^ India	Boys and girls 8–15 years OW/OB	Intervention: Intensive lifestyle intervention + yoga (*n* = 63) Control 1: Intensive lifestyle intervention (*n* = 59) Control 2: Waiting list: (*n* = 43)	1‐h session (5–10 min meditation) with yoga asanas and pranayama 5 times per week + energy restricted diet + 1 h moderate‐to‐vigorous exercise daily + behavioral modification for 18 weeks Same as intervention group, but without yoga No specific counseling	Yoga vs. controls: BMI, kg/m^2^: Yoga: −1.2 (CI: −2.3, −0.6) Control 1: −1.4 (CI: −3.1, −0.5) Control 2: 0.3 (CI: −0.3, 0.1) BMI, *z*‐score Yoga: −0.3 (CI: −0.4, −0.2) Standard: −0.4 (CI: −0.7, −0.2) Control: 0.0 (CI: −0.1, 0.1) *p *< 0.05 yoga and standard care against no intervention	67% yoga, 65% Control 1 and 65% Control 2 completed follow‐up

Abbreviations: BMI, body mass index; OB, obesity; OW, overweight.

One study offered one session per week of yoga plus physical activity and compared the results to the standard care offered. There was a decrease in BMI at the end of the intervention in the yoga group but an increase in the control group (−0.8 vs. 0.7 kg/m^2^, *p *< 0.001). BMI in the yoga group decreased at 6‐month follow‐up (−1.2 vs. 0.8 kg/m^2^, *p *< 0.001).^37^ A study that compared yoga plus intensive lifestyle interventions, including moderate‐to‐intense exercising, against two controls (one with a similar intervention except yoga, and the other without any intervention) found comparable decreases in BMI after yoga plus intensive lifestyle intervention (−1.2 kg/m^2^, IQR: −2.3, −0.6) and after intensive lifestyle intervention without yoga (−1.4 kg/m^2^, IQR −3.1, −0.5), but not in the no‐intervention group (0.3 kg/m^2^, 95% CI: −0.3, 0.1).^38^

We identified two other RCTs that were conducted in adolescents and used yoga to modify body weight.^39^, ^40^ Yoga was offered without any other intervention and was compared to no‐intervention groups in both studies; both reported significant improvements after yoga. In the first study, 50‐min yoga sessions, three times per week for 12 weeks, decreased BMI (−0.681 vs. −0.058 kg/m^2^) and fat mass (−2.879 vs. 0.200 kg) at the end of the intervention compared to controls (*p *< 0.001).^39^ The other study was conducted at home during the COVID‐19 pandemic to compare 45‐min online yoga sessions three times per week for 12 weeks compared to no intervention. Changes in BMI (23.14–21.96 kg/m^2^, *p* < 0.001) and waist circumference (28.25–25.5 cm, *p* < 0.001) at the end of follow‐up were observed.^40^ However, these studies were not included in our review because participants did not meet the selection criteria. The participants in both studies were 18–20 years of age, and those in the second study did not present with obesity (BMI between 23 and 26 kg/m^2^).

Three of the assessed studies analyzed the effect of yoga on psychological variables (Table 3). One identified improvements in eating behavior (e.g., emotional eating, external eating, and restrained eating) in the yoga condition compared to controls.^35^ Another did not observe any effects on stress, mood, or eating behavior.^37^ The third study detected improvements in stress, body shape, and quality of life scores only in the group that received intensive lifestyle recommendations without yoga.^38^ Two studies additionally examined the effect of yoga on selected metabolic variables (Table 3). One found decreases in total cholesterol and HDL‐cholesterol and increases in resting metabolic rate in the yoga group, but comparisons against controls did not reach statistical significance. HOMA‐IR did not change in the yoga group but did increase in the control group (*p* = 0.10).^36^ The other study detected significant improvements in systolic blood pressure after yoga intervention, whereas no effect on diastolic blood pressure or heart rate was detected in any group.^38^

**TABLE 3:** Effect of yoga on psychological and metabolic measurements of children and adolescents with obesity.

Author	Psychological outcomes	Metabolic and clinical outcomes
Sabet Sarvestani et al.^35^	From baseline to 6 months Emotional eating: Yoga: 1.89 to 1.29 Control: 1.87 to 2.18 Between‐group difference: *p *< 0.05 External eating: Yoga: 3.02 to 1.94 Control: 2.75 to 2.75 Between‐group difference: *p *< 0.05 Restrained eating: Yoga: 3.01 to 3.83 Control: 3.35 to 3.36 Between‐group difference: *p *< 0.05	
Seo et al.^36^		∆Yoga vs. ∆control Total cholesterol, mg/dL −14.30 vs. −11.90, *p* = 0.773 HDL cholesterol, mg/dL −27.70 vs. −21.90, *p* = 0.304 HOMA‐IR 0.16 vs. 5.54, *p* = 0.105 BMR, kcal/day 44.30 vs. 15.90, *p* = 0.195
Halperin et al.^37^	No effect on stress, mood, or eating behavior in any group	
Jain et al.^38^	Stress score Yoga: 19.2 ± 5.0 to 17.5 ± 5.8, *p *= NS Control 1: 20.1 ± 6.4 to 16.5 ± 5.3, *p *< 0.01 Control 2: 20.5 ± 7.6 to 17.2 ± 0.6, *p *= NS Body shape score Yoga: 46.0 ± 17.0 to 43.2 ± 16.6, *p *= NS Control 1: 42.8 ± 16.0 to 35.0 ± 13.9, *p* > 0.05 Control 2: 54.5 ± 15.5 to 48.1 ± 4.4, *p *= NS Quality of life score: Yoga: 84.8 ± 12.3 to 86.0 ± 13.2, *p *= NS Control 1: 85.2 ± 10.5 to 90.6 ± 10.4, *p *< 0.05 Control 2: 85.7 ± 14.2 to 86.1 ± 14.7, *p *= NS	Systolic blood pressure, mmHg Yoga: 118 ± 10 to 114 ± 8, *p *< 0.01 Control 1: 114 ± 9 to 116 ± 10, *p *= NS Control 2: 118 ± 9 to 116 ± 8, *p *= NS No changes in diastolic blood pressure or heart rate in any group

*Note*: Eating behavior was measured with the Dutch Eating Behaviour Questionnaire,^35^ or self‐reported via online questionnaires (SurveyMonkey).^37^ Stress, body shape, and quality of life scores were measured using psychological questionnaires (Cohen for perceived stress, Body Shape Questionnaire‐16, and Kindl R for assessing health‐related quality of life).

Abbreviations: BMI, body mass index; BMR, basal metabolic rate; HDL, high density lipoprotein; HOMA‐IR, Homeostatic Model Assessment for Insulin Resistance; NS, not significant; OB, obesity; OW, overweight.

Three of the four studies described detailed yoga sessions, which included short periods of relaxation/meditation for 3–10 minutes.^36^, ^37^, ^38^ The study that did not report offering meditation detected positive effects on anthropometric measurements and psychological variables.^35^ In two of the four studies all participants completed follow‐up assessments.^36^, ^37^ The attrition rate in the other two was 20%–35%. A study that analyzed a possible gender effect did not find an association between gender and changes in BMI (*p* = 0.87).^37^

In summary, all studies reported beneficial effects on body weight measurements after interventions with yoga plus additional physical exercise compared to no‐intervention controls. A study that compared yoga plus exercise against exercise alone failed to detect differences. Only one of the three studies that analyzed psychological variables reported positive effects. One of the two studies that assessed metabolic variables reported improvements in systolic blood pressure.

### Mindfulness

Seven RCTs on mindfulness for the management of obesity in children and adolescents were identified (Table 4).^41^, ^42^, ^43^, ^44^, ^45^, ^46^, ^47^ Two studies compared mindful eating against standard care. One compared family‐based mindful eating for 4 weeks against active controls (standard care in three 90‐min sessions). No differences between groups were found (mean difference: 0.6 kg/m^2^; Q1, Q3: −0.2, 1.2 vs. −0.3 kg/m^2^; −0.9, −0.1).^41^ The other study compared mindful eating for 6 weeks to one session of standard care. A greater decrease in BMI was reported in the mindful eating group compared to controls (intragroup difference: −1.1 ± 1.5 vs. 0.7 ± 1.0 kg/m^2^, *p *< 0.001).^42^

**TABLE 4:** Studies that analyzed the efficacy of mindfulness interventions for the management of children and adolescents with obesity.

Author country	Participants	Type of intervention (*n*)	Follow‐up	Anthropometric outcomes	Comments
Kumar et al.^41^ United States	Boys and girls 14–17 years BMI > 95th	Intervention: Mindful eating, family‐based (*n* = 11) Control: Dietary counseling (*n* = 11)	Four 90‐min sessions (Weeks 0, 1, 6, 10). No information about meditation. No dietary counseling Three 90‐min sessions (Weeks 0, 12, and 24)	ΔMND vs. Δcontrol: BMI, kg/m^2^: median (Q1, Q3) 0.6 (−0.2, 1.2) vs. −0.3 (−0.9, −0.1), *p* = ns BMI, *z*‐score: 0.1 (0, 0.2) vs. −0.1 (−0.2, 0), *p* = ns	82% MND and 100% controls completed follow‐up
Daly et al.^42^ United States	Girls 14–17 years BMI > 90th	Intervention: Mindful eating (*n* = 14) Control: Standard care (*n* = 23)	90‐min sessions, including 3 min meditation daily for 6 weeks One meeting for recommendations	ΔMND vs. Δcontrol at the end of intervention: BMI, kg/m^2^: −1.1 ± 1.5 vs. 0.7 ± 1.0, *p *< 0.001 ∆MND group 4 weeks post intervention: −1.4 kg/m^2^, *p* = 0.019	57% MND and 65% controls completed follow‐up
Barnes and Kristeller^43^ United States	Boys and girls 16.2 ± 1.2 years BMI 32 ± 9	Intervention: Mindful eating + mindfulness‐based stress reduction + health education + physical fitness (*n* = 18) Control: Health education + physical fitness (*n* = 22)	50‐min sessions of mindful eating, including mini meditation and some information of mindfulness‐based stress reduction once per week for 12 weeks 50 min sessions once per week for 12 weeks	MND vs. control: BMI, kg/m^2^: 32.9 ± 8.8 to 33 ± 8.9 vs. 32 ± 9.4 to 32.3 ± 9.8, *p *= NS	All completed follow‐up
Stavrou et al.^44^ Greece	Boys and girls 9–15 years BMI > 90th	Intervention: Mindfulness‐based stress reduction + low‐energy diet + physical activity (*n* = 37) Control: Diet + physical activity (*n* = 35)	1‐h session including meditation, once per week for 8 weeks + recorded sessions (at home) once per day. Attended separate sessions for standard care recommendations Unspecified number of sessions for standard care recommendations	ΔMND vs. Δcontrol: BMI, kg/m^2^: −1.2 ± 0.6 vs. −0.1 ± 0.6, *p* < 0.001	62% MND and 74% controls completed follow‐up
Emmanouil et al.^45^ Greece	Boys and girls 8–17 years BMI > 30	Intervention: Mindfulness‐based stress reduction + diet + physical activity training (*n* = 22) Control: Diet + physical activity training (*n* = 25)	1‐h sessions, including meditation once per week for 8 weeks + a CD to practice once a day at home + walking 60 min per day and sport activities 45–60 min three times per week Diet plus physical activity like intervention group	MND vs. controls: WHR: 0.92 (95% CI [0.89, 0.97]) to 0.89 (95% CI [0.85, 0.94]) vs. 0.94 (95% CI [0.89, 0.98]) to 0.97 (95% CI [0.89, 1), *p *= 0.008	73% MND and 80% controls completed follow‐up
Shomaker et al.^46^ United States	Boys and girls 12–17 years BMI > 70th plus depressive symptoms	Intervention: Mindfulness‐based stress reduction (*n* = 29) Control: Health education (*n* = 25)	1‐h sessions, including meditation once per week for 6 weeks, plus daily meditation at home 6 sessions of health education with videos, handouts, and presentations Evaluation at post‐intervention and at a 6‐month follow‐up	MND vs. controls Baseline to 6 weeks to 6 months BMI, kg/m^2^: 27.2 to 27.4 to 27.9 vs. 27.4 to 27.7 to 28.1, *p* = 0.91 Fat mass, %: 35.7 to 34.6 to 32.9 vs. 34.4 to 32.7 to 32.3, *p* = 0.64	93% MND and 76% controls completed 6‐week follow‐up
López‐Alarcón et al.^47^ Mexico	Boys and girls 10–14 years BMI > 95th plus anxiety	Intervention: Mindfulness‐based stress reduction + standard care (*n* = 47) Control: Standard care (*n* = 16)	2‐h sessions, including meditation once per week for 8 weeks + daily meditation at home. Parallel parental sessions 1‐h sessions once per week with parents for diet and physical activity recommendations Evaluation at 8 and 16 weeks	ΔMND vs. Δcontrol End of intervention (8 weeks) BMI, kg/m^2^: −0.12 ± 0.16 vs. −0.05 ± 0.09, *p* = 0.081 Fat mass, %: −1.28 ± 0.25% vs. −1.24 ± 0.91, *p* = 0.527 MND group, ΔBMI: 8‐week: −0.12 ± 0.04, *p* = 0.004 16‐week: −0.17 ± 0.05, *p* = 0.004	70% MND and 75% controls completed intervention

Abbreviations: BMI, body mass index; MND, mindfulness; WHR, waist‐to‐hip ratio.

Four studies assessed mindfulness‐based stress reduction combined with some additional strategy, such as a low‐energy diet and physical activity recommendations,^44^, ^47^ diet and physical activity training,^45^ or a health education program.^46^ These results were compared to an active control group that received the additional strategy as the intervention (e.g., low energy and physical activity recommendations, diet and physical activity training, or a health education program). All four studies reported favorable effects of mindfulness in at least one anthropometric measurement. One of these studies detected a higher decrease in BMI compared to controls (−1.18 ± 0.62 vs. −0.10 ± 0.56 kg/m^2^, *p *< 0.001).^44^ Emmanouil et al. reported a reduction of waist‐to‐hip ratio in the mindfulness condition compared to increases in the control group (0.92–0.89 vs. 0.94–0.97; *p* = 0.008).^45^ Another study found reductions of absolute and relative fat mass after the intervention, but the results were similar between the mindfulness and health education groups (35.66 ± 1.41 to 34.63 ± 1.38 kg vs. 34.38 ± 1.52 to 32.71 ± 1.48 kg).^46^ One study observed decreases in BMI (−0.12 ± 0.16 kg/m^2^, *p* = 0.009), BMI *z*‐score (−0.07 ± 0.03, *p* = 0.008), and fat mass (−1.24% ± 0.25%, *p* < 0.001) in mindfulness group, whereas no changes were observed in the control group (between groups difference *p* = 0.081).^47^ Two studies analyzed the effect of mindfulness 5 and 8 weeks after finishing the intervention period. In the first study, fat mass continued decreasing at 6 months, but it was similar in both groups.^46^ In the other study, BMI continued decreasing in the mindfulness group after 8 weeks post‐intervention (difference from baseline: −0.17 ± 0.05 kg/m^2^, *p* = 0.004), but the control group did not attend the follow‐up after the intervention period.^47^

Another study assessed the effect of mindful eating combined with mindfulness‐based stress reduction. This treatment was compared to a health education program. Both groups were enrolled in physical fitness classes. Neither group showed changes from baseline to the end of the intervention (BMI: 32.9 ± 8.8 to 33 ± 8.9 kg/m^2^ vs. 32 ± 9.4 to 32.3 ± 9.8 kg/m^2^ for mindfulness and control groups, respectively). All participants completed the follow‐up.^43^ The risk of bias was unclear for all the components (Table 1).

Six of the seven reviewed RCTs assessed the effect of mindfulness on psychological variables (Table 5)^41^, ^43^, ^44^, ^45^, ^46^, ^47^; four found positive effects.^43^, ^44^, ^46^, ^47^ Two of these studies included depression^46^ or anxiety^47^ as criteria to enter the study to ensure all participants presented a psychological symptom before intervention. In one of the studies, there were improvements in perceived stress (*p* < 0.05), food reward sensitivity (*p* < 0.05), stress eating (*p* < 0.01), and emotional control (*p* = 0.08) in the intervention group.^46^ The other study reported reductions in anxiety scores in the mindfulness group while increases were observed in controls (−6.21 ± 1.10 vs. 0.66 ± 0.64; *p* < 0.001).^47^ One study reported greater improvements in anxiety/depression and in internalizing/externalizing problems in the mindfulness group compared to controls,^44^ whereas another study did not find effects on anxiety, depression, or aggressive behavior in either group.^45^ It is important to note that this study did not report the proportion of children with psychological symptoms at baseline.

**TABLE 5:** Effect of mindfulness in psychological and metabolic measurements of children and adolescents with obesity.

Author	Psychological outcomes	Metabolic outcomes
Kumar et al.^41^	Negative emotions, social pressure, and physical discomfort did not change in any group	∆MND vs. ∆control HDL cholesterol, median (Q1, Q3): 7 (2, 10) vs. −1 (−2, 3), *p* = 0.024 Glucose, insulin, cholesterol, triglycerides, and C‐reactive protein did not change in MND but increased in controls. There were no between‐group differences
Barnes and Kristeller^43^	∆MND vs. ∆controls: Perceived hunger: 5.6 ± 3 to 3.4 ± 1.8 vs. 5.8 ± 3.6 to 4.8 ± 3.2, *p* = 0.10 Binge eating scale: 9.9 ± 7.2 to 11 ± 7 vs. 15 ± 10 to 10.6 ± 10.9, *p* < 0.01 Anger expression: 8.5 ± 8.6 to 11.5 ± 8.5 vs. 11 ± 9 to 10.6 ± 10.9, *p* = 0.10	
Stavrou et al.^44^	∆MND vs. ∆control: Anxious/Depressed: −1 ± 5.4 vs. 3.5 ± 3.6, *p* = 0.001 Internalizing problems: −4.4 ± 7.5 vs. 5.7 ± 4.8, *p* < 0.001 Externalizing problems: −1.9 ± 6.5 vs. 4.6 ± 3.5, *p* < 0.001	
Emmanouil et al.^45^	No changes in psychological variables (stress and anxiety scores) in any group	CAR (µg/dL) 6.07 × 10^−3^ to 5.69 × 10^−3^ vs. 6.77 × 10^−3^ to 4.37 × 10^−3^, *p* = 0.484
Shomaker et al.^46^	∆MND vs. ∆control: Six weeks Perceived stress: −0.36 vs. 4.63, *p* = 0.09 Six months Perceived stress: −5.56 vs. 0.09, *p* = 0.06 Food reward sensitivity: 2.63 vs. 22.53, *p* = 0.01 Stress eating, kcal: −2.33 vs. 276, *p* = 0.05 Emotional control: −0.19 vs. 3.24, *p* = 0.08	
López‐Alarcón et al.^47^	∆MND vs. ∆control: Panic disorder: −1.96 ± 0.76 vs. 0.58 ± 0.22, *p* = 0.020 Anxiety disorder: −2.21 ± 0.37 vs. 0.08 ± 0.41, *p* = 0.002 Generalized anxiety disorder: −2.18 ± 0.56 vs. 0 ± 0.27, *p* = 0.011	∆MND vs. ∆control: Cortisol (µg/dL): −1.42 ± 0.94 vs. 2.26 ± 0.93, *p* = 0.015 Leptin (ng/mL): 0.19 ± 0.64 vs. −0.68 ± 1.18, *p* = 0.271 Ghrelin (pg/mL): −0.71 ± 0.37 vs. 0.83 ± 0.75, *p* = 0.026

*Note*: CAR is the surge in cortisol production that occurs shortly after waking in the morning. It calculated as the difference between the 30–45 min post‐awakening cortisol (C2) and awakening cortisol (C1) divided by the mean time elapsed between these two measurements, that is, [(C2 − C1)/45].

Abbreviations: CAR, cortisol‐awakening response; HDL, high‐density lipoproteins in mg/dL; MND, mindfulness.

Five of the six studies that analyzed psychological symptoms used the mindfulness‐based stress reduction technique.^43^, ^44^, ^45^, ^46^, ^47^ Only one of these studies did not find any effect.^45^ One study that used mindful eating did not observe effects on psychological symptoms nor BMI.^41^

Three RCTs additionally explored the effect on metabolic outcomes (Table 5). Increases of HDL‐c concentration in mindfulness but not controls conditions (7; 2, 10 mg/mL vs. −1; −2, 3 mg/mL; *p* = 0.024) were identified in one study despite no changes in weight measurements.^41^ A study that observed the effects on waist‐to‐hip ratio, but not in BMI, failed to identify effects of mindfulness in the cortisol‐awakening response (*p* = 0.484).^45^ Decreases in serum ghrelin (−0.71 ± 0.37 vs. 0.83 ± 0.75 pg/mL; *p* = 0.026) and cortisol (−1.42 ± 0.94 vs. 2.26 ± 0.93 µg/dL; *p* = 0.015) after mindfulness were reported in another study that also found effects on anthropometric measurements.^47^

All participants completed the follow‐up in one study^43^; the attrition rate in the other studies ranged between 3% and 43%. One study assessed the probability of a gender effect for binge eating but not for body weight; no effect was identified.^43^ None of the studies reported adverse events associated with the mindfulness interventions. However, as in yoga studies, it is unclear whether adverse events did not occur or were not assessed.

In summary, five of seven studies identified positive effects of mindfulness on body weight measurements compared to active controls; one study detected decreases in fat mass similar to changes observed in controls. Four of the six studies that analyzed psychological symptoms detected positive effects on stress, anxiety, depression, and eating behavior. Two out of three trials that analyzed metabolic variables found positive effects.

## DISCUSSION

The assessment of the likely mechanisms used by yoga and mindfulness to impact obesity in children and adolescents suggests that an effect is plausible. However, most of the identified studies had a high risk of bias and serious methodological shortcomings. Therefore, the available evidence is insufficient to establish the efficacy of these interventions.

Studies of yoga conducted in children with obesity are scarce and not methodologically robust. A systematic review of eight studies (four one‐arm and four RCTs) reported inconsistent results. Two one‐arm and two RCTs detected significant pre/post intervention changes in weight or BMI; only one RCT found differences with the control group.^48^ In our review, we found that all the assessed RCTs reported reductions in BMI. However, such effects could not be ascribed to yoga alone because it was combined with additional physical exercise other than yoga, and the additional physical exercises were not offered as controls. Interestingly, in contrast to studies in children, studies in adults consistently show that yoga may be useful for the management of obesity and provide some evidence on the specific role of meditation added to the energy expenditure derived from the physical components of yoga. A meta‐analysis of RCTs that compared the effect of yoga against other physical exercise intervention in individuals with obesity reported an extra benefit of yoga over physical exercise on BMI (*Δ*: −0.71 kg/m^2^, 95% CI [−1.28, −0.15]).^30^ Another meta‐analysis of RCTs that compared yoga against no intervention, calorie restriction, and physical exercise found that body weight decreased more in yoga groups if compared to calorie restriction only (*d*: −3.47 kg, 95% CI [−6.20, −0.74]) or physical exercise only (*Δ*: −7.58 kg, 95% CI [−11.51, −3.65]).^49^ Articles included in these meta‐analyses were of low methodological quality. However, they consistently suggest the effectiveness of yoga to impact body weight through both increasing energy expenditure and meditation.

In our study, the potential beneficial effect of yoga meditation could not be evaluated because only short periods of relaxation (unclear if it could be considered meditation) were described in three of the four reviewed studies, and the one that did not describe the details of yoga sessions found effects on BMI comparable to those reported in the other three studies.^35^ On the other hand, the potential effect through increasing energy expenditure could not be evaluated either because moderate‐to‐intense physical activity was included as an additional intervention to yoga in all the studies. The study of Jain et al. found that BMI decreased similarly in the two groups that received intense physical activity, with or without yoga,^38^ suggesting that physical activity is the main mechanism through which yoga might impact body weight in children. This hypothesis is reinforced by the two studies by Na Nongkhai et al. that reported significant effects of yoga on body weight because interventions were designed to focus on the physical aspect of yoga practice by using minimal resting periods and trying to reach the maximal aerobic capacity to obtain a workout sufficient to increase energy metabolism.^39^, ^40^

Studies of mindfulness in adolescents are contradictory. A 1‐arm study conducted in 11 adolescents with obesity found no effect in BMI after 6 weeks of mindfulness intervention.^50^ In contrast, a study in youths (not included in our review because it also involved adults) found that those with high levels of stress‐eating or compulsive‐eating lost more weight after mindfulness intervention than controls at a 1‐year follow‐up.^51^ In our review, five out of seven trials reported positive effects on at least one anthropometric variable, despite all but one study comparing mindfulness to active controls. These results suggest an advantage of mindfulness over other types of cognitive interventions, but this needs to be demonstrated. In adults, the effect of mindfulness on anthropometric measurements also remains unclear.

Multiple studies have consistently found beneficial effects of mindfulness on stress and sedentary and eating behaviors in individuals with obesity. However, it has not been established that such effects impact body weight.^52^, ^53^, ^54^, ^55^, ^56^ A systematic review of six RCTs found favorable effects on emotional eating and eating behavior in comparison to no intervention or cognitive–behavioral therapies. However, changes in body weight were only found in one study that compared mindfulness to no intervention.^53^ Likewise, three meta‐analyses of RCTs reported positive effects of mindfulness intervention on eating and physical activity behaviors, or stress, but not in body weight.^54^, ^55^, ^56^ Most of the studies in these reviews were of low quality, had high heterogeneity, and had a high risk of bias. In contrast, two other meta‐analyses with better quality reported significant effects of mindfulness to decrease body weight. One of these meta‐analyses of RCTs that assessed the effect of mindful eating on obesity concluded that it was useful to decrease body weight compared with a no‐intervention group, but not if compared with active controls.^56^ The other meta‐analysis, with small heterogeneity and low risk of bias, observed improvements in BMI, body weight, and waist circumference similar to improvements provided by dietary interventions. Four RCTs that included post‐intervention assessments reported a sustained effect at week 15. Unfortunately, stress and eating or sedentary behaviors were not assessed in these studies. Yet, these results suggest a beneficial effect of mindfulness on excess body weight, at least similar to the effect of other interventions.^57^

A topic that merits discussion is the type of mindfulness intervention. The more frequently used mindfulness techniques for obesity management in scientific literature are mindfulness‐based stress reduction^58^ and mindful eating,^59^ but these techniques have important differences. Although the first is expected to improve mental health in individuals with chronic physical health problems related to stress,^60^ the second is used to train individuals for a state of consciousness focused on food for a non‐judgmental awareness of internal and external cues that influence food choice, the desire to eat, and the quality of foods.^61^ Importantly, mindful eating uses short periods of meditation within sessions, whereas mindfulness‐based stress reduction uses long periods of meditation within a session in addition to meditation at home. Based on these findings, it would be expected that there would be a major influence of mindfulness‐based stress reduction than mindful eating. In our systematic review, one of the studies that used mindful eating did not find effects on anthropometric measurements. However, another study did detect an effect on BMI of an equal magnitude to that observed in the studies that used mindfulness‐based stress reduction. On the other hand, one study that used mindfulness‐based stress reduction failed to detect changes in body weight measurements. Therefore, we cannot draw any conclusions regarding a differential effect between either of these interventions.

If the effect of mindfulness on body weight is mediated through improvements in stress of an individual, then a correlation between changes in stress and changes in BMI would be expected. This was demonstrated in a study conducted in women with obesity and stress who underwent a mindfulness intervention. Changes in the cortisol awakening response after the intervention correlated positively with changes in BMI (*r* = 0.57, *p* = 0.02).^62^ This association could not be assessed in our review. Although three studies detected improvements in both psychological manifestations and weight measurement, one found improvements in eating‐related variables but not in anthropometric measurements; none of the studies assessed the correlation between changes in psychological symptoms and changes in anthropometric outcomes.

Thus, there is insufficient evidence to demonstrate the efficacy of meditation‐based interventions such as yoga and mindfulness in the management of children and adolescents with obesity, mainly because of the methodological weaknesses of the available studies. However, in addition to the quality of the studies, it is important to consider that the success of these interventions depends on several characteristics of the interventions themselves that are difficult to account for (e.g., the components and intensity of interventions, the skills of instructors, family engagement, adherence to interventions at home, time of exposure, and sample size, among others). For instance, usually the number of individuals participating in these studies is ideally small because each participant needs to receive special assistance to ensure yoga movements or mindfulness are performed correctly (e.g., posture, breathing, and focusing). Moreover, mindfulness interventions are conceived to be short in length (6 weeks for mindful eating, 8 weeks for mindfulness‐based stress reduction), which hampers the possibility of seeing effects in the long term, particularly because effects on body weight may take longer to manifest. These methodological features may prevent the detection of an effect on weight if it exists. Nevertheless, the underlying mechanisms and results from this review are a starting point for planning future research assessing patient‐relevant outcomes and addressing the limitations identified for the studies included in this work.

Finally, none of the reviewed studies reported adverse events associated with the interventions. However, it is unclear if the authors did not assess the presence of adverse events or if they did not occur. Thus, specific studies designed to analyze the safety of these interventions need to be planned.

## CONCLUSIONS

There is insufficient evidence on the efficacy and safety of yoga and mindfulness as therapies for the management of children and adolescents with obesity.

## AUTHOR CONTRIBUTIONS

Mardia López‐Alarcón and José R. Fernández conceived the study. Mardia López‐Alarcón and Miguel A. Villasis‐Keever designed the study protocol and performed data analysis. Miguel A. Villasis‐Keever executed literature search. Mardia López‐Alarcón and Miguel A. Villasis‐Keever screened the studies and analyzed data. Mardia López‐Alarcón, Miguel A. Villasis‐Keever, and José R. Fernández interpreted the results. All authors edited, revised, and approved the final version of the manuscript.

## CONFLICT OF INTEREST STATEMENT

The authors declare no conflicts of interest.

## DISCLAIMER

This manuscript was developed as input for the World Health Organization project “Integrated management of children and adolescents with obesity in all their diversity: A primary health care approach for improved health, functioning, and reduced obesity‐associated disability.” This article is being published individually but will be consolidated with other manuscripts as a special issue of *Annals of the New York Academy of Sciences*; the coordinators were Maria Nieves Garcia‐Casal and Hector Pardo‐Hernandez. This special issue will provide insights to support the World Health Organization in developing practice and science‐informed, people‐centered guidelines on the integrated management of children 0–9 and adolescents 10–19 years of age in all their diversity with obesity using a primary health care approach. The special issue is the responsibility of the editorial staff of *Annals of the New York Academy of Sciences*, who delegated to the coordinator's preliminary supervision of both technical conformity to the publishing requirements of *Annals of the New York Academy of Sciences* and general oversight of the scientific merit of each article. The findings and conclusions in this article are those of the authors and do not necessarily represent the official position of the World Health Organization, the publisher, or editorial staff of *Annals of the New York Academy of Sciences*.

### PEER REVIEW

The peer review history for this article is available at: https://publons.com/publon/10.1111/nyas.15245

## Supporting information



Supporting Information S1. PubMed strategy search.

Supporting Information S2. EMBASE strategy search.

Supporting Information S3. APA PsycInfo strategy search.
