# Supplementary material for: Systematic review of the efficacy of yoga and mindfulness in the management of pediatric obesity
Source: Ann N Y Acad Sci. 2024 Dec 19;1543(1):17–30. doi: 10.1111/nyas.15245 (PMC11776448; doi:10.1111/nyas.15245)
Supplement: Supplementary file 1 — Supporting Information S1. PubMed strategy search. [file NYAS-1543-17-s003.docx]

**Supporting Information 1**

**PUBMED strategy search**

**Date: March 31^st^, 2024**

("mindfulness*"[MeSH Terms] OR "mindfulness based stress reduction"[Text Word] OR "MBSR"[Text Word] OR "mindfulness based eating awareness"[Text Word] OR "mindful eating"[Text Word] OR "meditation"[MeSH Terms] OR "Mindfulness"[Text Word] OR "mindful eating"[Text Word] OR "behavior therapy"[MeSH Terms] OR "yoga*"[MeSH Terms] OR (("Yoga"[MeSH Terms] OR "Yoga"[All Fields]) AND "psychology*"[MeSH Terms]) OR "Yoga"[Text Word]) AND ("Obesity"[MeSH Terms] OR "weight gain"[MeSH Terms] OR (("obeses"[All Fields] OR "Obesity"[MeSH Terms] OR "Obesity"[All Fields] OR "Obese"[All Fields] OR "obesities"[All Fields] OR "obesity s"[All Fields]) AND "psychology*"[MeSH Terms]) OR ("pediatric obesity/prevention and control"[MeSH Terms] AND "control*"[Text Word]) OR "pediatric obesity/psychology"[MeSH Terms] OR "overweight/psychology"[MeSH Terms] OR (("Overweight"[MeSH Terms] OR "Overweight"[All Fields] OR "overweighted"[All Fields] OR "overweightness"[All Fields] OR "overweights"[All Fields]) AND "therapy*"[MeSH Terms]) OR "Obese"[Text Word] OR "Obesity"[Text Word]) AND ("clinical trial"[Publication Type] OR "adaptive clinical trial"[Publication Type] OR "clinical trial, phase i"[Publication Type] OR "clinical trial, phase ii"[Publication Type] OR "clinical trial, phase iii"[Publication Type] OR "clinical trial, phase iv"[Publication Type] OR "controlled clinical trial"[Publication Type] OR "pilot projects"[MeSH Terms] OR "pilot study"[Text Word] OR "randomized controlled trial"[Publication Type] OR "clinical trial*"[Text Word] OR "clinical study"[Publication Type] OR "randomized controlled trial"[Text Word])
