# Supplementary material for: Systematic review of the efficacy of yoga and mindfulness in the management of pediatric obesity
Source: Ann N Y Acad Sci. 2024 Dec 19;1543(1):17–30. doi: 10.1111/nyas.15245 (PMC11776448; doi:10.1111/nyas.15245)
Supplement: Supplementary file 2 — Supporting Information S2. EMBASE strategy search. [file NYAS-1543-17-s001.docx]

**Supporting Information 2**

**EMBASE search strategy**

**Date: March 31^st^, 2024**

(('mindfulness meditation'/exp OR 'mindfulness meditation') OR 'mindfulness-based stress reduction' OR 'mindful eating' OR 'mindfulness' OR 'yoga') AND ('obesity' OR 'body weight gain' OR 'obese patient' OR 'obesity psychology' OR 'childhood obesity'/exp)
