# Supplementary material for: Systematic review of the efficacy of yoga and mindfulness in the management of pediatric obesity
Source: Ann N Y Acad Sci. 2024 Dec 19;1543(1):17–30. doi: 10.1111/nyas.15245 (PMC11776448; doi:10.1111/nyas.15245)
Supplement: Supplementary file 3 — Supporting Information S3. APA PsycInfo strategy search. [file NYAS-1543-17-s002.docx]

**Supporting Information S3**

**APA PsycInfo <1806 to May Week 5 2024>**

(Mindfulness/ or Mindfulness-Based Interventions/ or Mindfulness-based stress reduction.mp. [mp=title, abstract, heading word, table of contents, key concepts, original title, tests & measures, mesh word] **OR** (MBSR or Mindfulness-Based Eating Awareness or Mindful eating or meditation).mp. [mp=title, abstract, heading word, table of contents, key concepts, original title, tests & measures, mesh word] **OR** (Mindfulness or Mindful Eating or Behavior Therapy or Yoga or Physical Activity or YOGA).mp. [mp=title, abstract, heading word, table of contents, key concepts, original title, tests & measures, mesh word])

**AND**

(Obesity or Weight Gain or Obesity psychology).mp. [mp=title, abstract, heading word, table of contents, key concepts, original title, tests & measures, mesh word] **OR** (Pediatric Obesity or (Obesity or Pediatric Obesity) or prevention & control).mp. [mp=title, abstract, heading word, table of contents, key concepts, original title, tests & measures, mesh word] **OR** (Pediatric Obesity psychology or (Overweight or "Overweight and obesity")).mp. [mp=title, abstract, heading word, table of contents, key concepts, original title, tests & measures, mesh word] **OR**  (Obese or obesity).mp. [mp=title, abstract, heading word, table of contents, key concepts, original title, tests & measures, mesh word]

**AND**

*Randomized Clinical Trials/ or *Clinical Trials/ or *Randomized Controlled Trials/ or (Clinical Trial Type or Clinical trial participant person or Clinical Trial Phase II or Controlled Clinical Trial Publication Type or Clinical Trials or Phase I Clinical Trials or Phase 2 Clinical Trials or Phase 4 Clinical Trials or Clinical Trials Randomized).mp. **OR** (Pilot Projects or pilot study or Clinical Research or clinical study).mp. [mp=title, abstract, heading word, table of contents, key concepts, original title, tests & measures, mesh word]
